# Low-Cost Oil Quality Sensor Based on Changes in Complex Permittivity

**DOI:** 10.3390/s111110675

**Published:** 2011-11-10

**Authors:** Angel Torres Pérez, Mark Hadfield

**Affiliations:** Sustainable Design Research Centre, Bournemouth University, Fern Barrow, Talbot Campus, Poole, Dorset, BH12 5BB, UK; E-Mail: mhadfield@bournemouth.ac.uk

**Keywords:** oil quality, impedance spectroscopy, marginal oscillator, dielectric, permittivity, lubrication oil, on-line monitoring

## Abstract

Real time oil quality monitoring techniques help to protect important industry assets, minimize downtime and reduce maintenance costs. The measurement of a lubricant’s complex permittivity is an effective indicator of the oil degradation process and it can be useful in condition based maintenance (CBM) to select the most adequate oil replacement maintenance schedules. A discussion of the working principles of an oil quality sensor based on a marginal oscillator to monitor the losses of the dielectric at high frequencies (>1 MHz) is presented. An electronic design procedure is covered which results in a low cost, effective and ruggedized sensor implementation suitable for use in harsh environments.

## 1. Introduction

Oil quality sensors provide an indication of the condition of oils by measuring different fluid characteristics such as viscosity, density, optical (light scattering) and electrical properties (permittivity and conductance).

Viscosity is an important indicator of oil condition because it changes abruptly when there is a lubricant breakdown. There are several sensing techniques for performing viscosity and density measurements. However, most common types of commercially available process rheometers rely on resonators [1–10]. Resonator measurement principles are based on changes in the resonant frequency and the damping or Q factor. If the mechanical structure of the resonator is brought into contact with a fluid or solid medium both resonance frequency and damping are changed depending on the viscosity and the elasticity of the fluid. A recent review of methods for on-line monitoring of viscosity of lubrication oils is reported by [11].

Light scattering oil quality sensors rely on spectrometric techniques such as infrared, fluorescence and Raman spectrometry. The most effective indication of oil condition requires a calibration process using reference oil sample spectra and regression data analysis to isolate the influence of contaminants within the spectra [12]. Commercial implementations vary from the simplest and lowest cost sensor which only monitors the light absorbance at a few frequencies [13], whilst the most complex ones implement high resolution interferometric spectrometers.

An indication of the condition of oil can be extracted from the fluid electrical properties. In the current market, several types of oil quality sensors are available based on conductivity and permittivity measurements at one frequency. The conductivity ones are based on potentiostat measurements. The electrodes can be based on a polymeric bead matrix structure in which the detection principles are based on changes to the resistance of the polymer that depend on oxidation products and free water [14], or electrodes made with dissimilar metals where the potential difference between the sensitive and reference electrodes can be detected (pH probe) [15] or detecting the point when the lubricant starts to conduct applying an specific voltage waveform to the electrodes and using current to voltage converters [16,17]. A conductivity sensor for monitoring degradation of automotive engine oil based on polymers is studied in [18].

The sensors based on permittivity measurements are classified in two types depending on output. The first ones only monitor changes in the real part of the permittivity and the output of the second ones is related to the complex permittivity. The ones that monitor the real part of the permittivity measure changes in the capacitance of the electrodes whilst the sensors that monitor the complex permittivity provide output related to the capacitance and dielectric losses. The parameter that relates these two quantities of the complex permittivity is the dissipation factor (D or tan δ) which is the ratio between the imaginary part and the real part. The real part of the permittivity can be measured using low cost electrodes [19] and very simple circuits such as bridges [20,21], resonant circuits [22], astable multivibrators [23].

All measurement techniques for impedance spectroscopy (IS) are suitable to characterise the dissipation factor for a broad range of frequencies. For frequencies less than 100 KHz, a voltage bridge feed by a stable oscillator [24] or the use of an autobalancing bridge method [25] is typically used. A more detailed description of impedance measurement methods is covered in [26]. This research presents a low cost method to monitor the oil quality by monitoring changes of the complex permittivity (tan *δ*) at high frequencies (>1 MHz).

**Figure 1.**
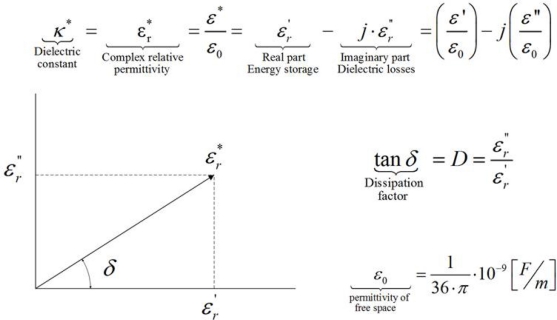
Definition of complex relative permittivity [26].

The complex permittivity of lubrication oils changes with use, mainly because of the process of oxidation and degradation of additives. This process is affected by the presence of contaminants such as water, soot particles, acid combustion products, glycol, ferrous and non-ferrous metallic particles. The degradation of most oils imply the generation of molecules that are generally more polar than the previous ones. The base oil consists of large hydrocarbon molecules that are generally weakly polar, so the presence of most contaminants results in an increase of one or both parts of the oil’s complex permittivity [27]. Depending on the geometry of the electrodes, the complex permittivity is directly related to complex impedance. For simple electrode geometries such as parallel plate and cylindrical electrodes, the relationship between complex permittivity, capacitance and complex impedance *vs.* frequency is analytically obtained. Expressions for the capacitance of parallel plate and cylindrical capacitors are shown in Table 1. Using the concept of complex relative permittivity, Figure 1, the complex impedance of a capacitor with losses (C*) can be determined using Equation (1):

(1) $$Z=\frac{1}{j\omega \cdot C*}$$

**Figure 2.**
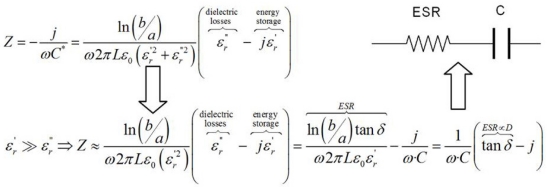
Equivalent circuit for cylindrical electrodes

The complex capacitance depends on the electrodes geometry and it corresponds to the same expressions shown in Table 1 using the complex permittivity. The final complex impedance expression is a function of the sensing electrodes geometry, excitation frequency and the condition of the oil which is related to complex permittivity. For the cylindrical geometry the procedure for the impedance determination is shown in Figure 2.

Lubricating oil is a dielectric material with low losses (it is a good electrical insulator as it has low conductivity). Therefore, the dissipation factor for oil can be considered much lower than unity and hence, the real part of the complex permittivity is higher than the imaginary part. The complex impedance expression suggests a simple circuit for the cylindrical electrode system as shown in Figure 2. This equivalent circuit consists of one capacitor and one resistor connected in series (equivalent series resistor, ESR). The equivalent circuit helps to understand the influence of the real and imaginary parts of the complex permittivity in the final impedance expression. The real part of the permittivity is related to the energy storage and the imaginary part to dielectric losses.

As a result, any measurement of complex impedance of the sensing electrodes is an indicator of the degradation of the oils. In analytical chemistry the measurement technique of electrode impedance as a function of frequency is commonly referred to as Electrochemical Impedance Spectroscopy (EIS). The underlying idea of Impedance Spectroscopy (IS) is the measurement and characterization of a material-electrode system. A complete impedance spectroscopy analysis involves more than a single set of measurements of impedance *vs.* frequency. Frequently, full characterization requires that such sets of measurements are carried out over a range of temperatures and/or other externally controlled experimental variables [28].

**Figure 3.**
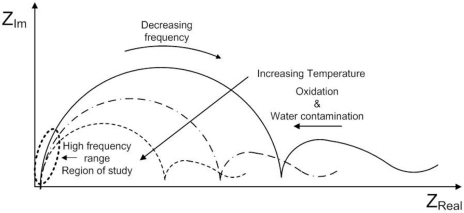
Nyquist diagram showing the influence of temperature and contamination in the impedance.

During the last decade, several research studies considering the application of electrochemical impedance spectroscopy in lubrication have appeared [29–34]. Important conclusions are drawn from these studies; the impedance response is dependent on the electrode’s geometry and its contact with the medium. A diagram of the effects in impedance of temperature, oxidation and water contamination [29–34] is shown in Figure 3.

It is concluded from the electrochemical studies and patents of impedance measurements of lubrication oils [29–34] and the Nyquist plots that low frequencies are the most sensitive to variations in impedance due to changes of the dielectric constant of the fluid. However, the shortcoming of performing low frequency impedance measurements is that small deviations of the measurement frequency lead also to great changes in impedance. Yet, high frequency impedance measurements provide similar readings of impedance at the vicinities of the nominal measurement frequency. However, high frequency impedance measurements provide lower sensitivity to detect changes in the dielectric constant of the fluid under test. Finally, this paper discusses the design of a low cost and effective oil quality circuit capable to monitor changes in the real part of the impedance of the sensing electrodes at high frequencies.

## 2. Sensor Design

### 2.1. Operation Principles

**Figure 4.**
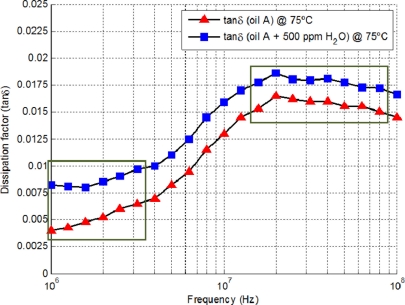
tan δ sweep for a lubricant without water contamination and contaminated with water.

The range of complex permittivity for different lubricant degradation stages can be obtained using an impedance analyzer performing impedance frequency sweeps. According to Collister [27], the values of the dissipation factor (tan δ) are between 0.001 and 0.1 within 1–300 MHz (0.001 for an unused mineral oil and 0.1 for a heavily contaminated sample) and the real permittivity is typically 2.25 (unused oil) to 2.45 respectively $\left({{ɛ}^{\prime }}_{r}\gg {{ɛ}^{\prime \prime }}_{r}\right)$. A frequency sweep example of the dissipation factor for lubricant at 75 °C without and with water contamination is shown in Figure 4. As it can be seen, the dissipation factor increases with water contamination. At lower frequencies the increment in dissipation factor for the oil without and with oil contamination is slightly larger than at higher frequencies.

**Figure 5.**
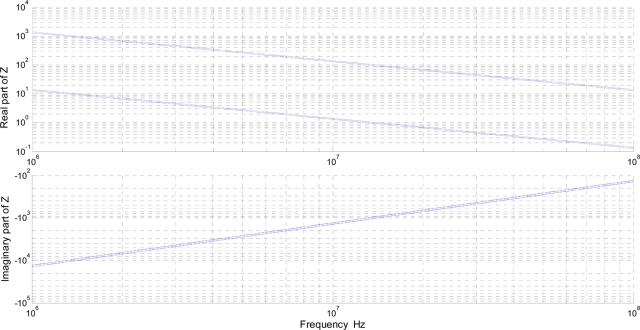
Operational range of impedance for the sensor (cylindrical head).

The working range of impedance, Figure 5, is obtained using the expected range of complex permittivity and the geometrical dimensions of the sensor’s cylindrical head (Table 2). The real part of Z (ESR) is within the range of 1 to 1 kΩ within this frequency range. The impedance chart of Figure 5 highlights the real part of Z varies in a larger range (2 decades) than the imaginary part (low range of values). Any circuit which monitors the real part of the impedance is more sensitive than a circuit which only measures the imaginary part. Therefore, any method to monitor the degradation of oils using capacitive measurements (imaginary part of the impedance) is not as effective as methods that monitor the dielectric losses.

### 2.2. Circuit Design

**Figure 6.**
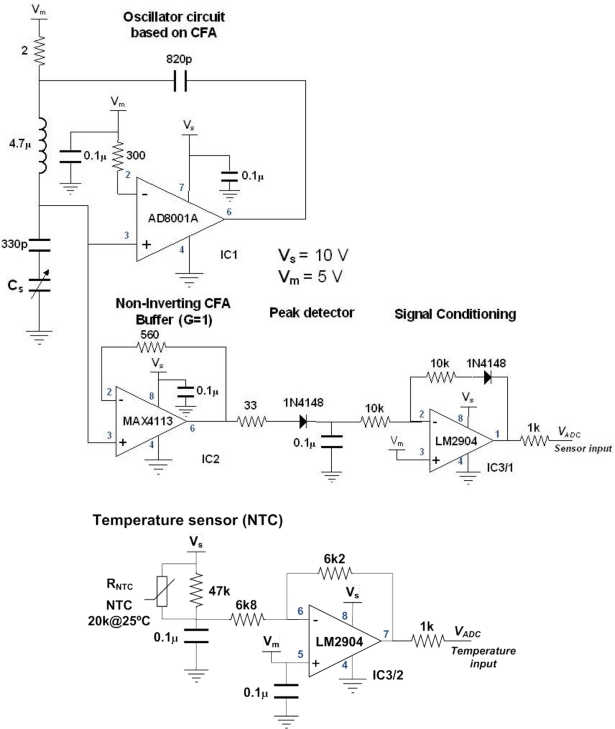
Hardware schematic (analog part).

The hardware schematic of the sensing part is shown in Figure 6. The complete sensor comprises a sensing head which is a cylindrical capacitor made of copper with geometrical dimensions as shown in Table 2, an LC oscillator circuit implemented with a current feedback amplifier (CFA), an oil temperature sensor embedded in the sensor’s head (NTC thermistor), some signal conditioning stages and one microcontroller (MCU). The MCU performs ADC conversions and it implements the temperature and calibration functions. As an option, the MCU supports industrial communications protocols such as RS232/RS485 and 4–20 mA.

#### 2.2.1. Oil Temperature Sensing Circuit

Oil temperature is measured using an NTC thermistor embedded in the sensor head that is contacting the inner electrode of the cylindrical capacitor (the outer electrode is grounded). The selected thermistor has a resistance of 20 kΩ at 25 °C and its resistance is adjusted according to the regression model proposed by Steinhart, Equation (2).

(2) $$\frac{1}{T}=b_{0}+b_{1}\cdot {\mathrm{lnR}}_{T}+b_{3}\cdot {\left[{\mathrm{lnR}}_{T}\right]}^{3}$$

**Figure 7.**
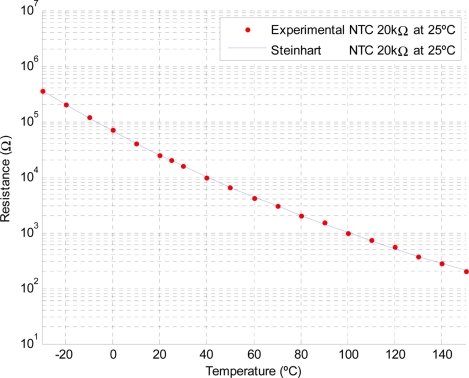
Thermistor resistance *vs.* temperature.

Figure 7 shows a plot of the NTC experimental resistance and the proposed Steinhart model according to Table 3.

**Figure 8.**
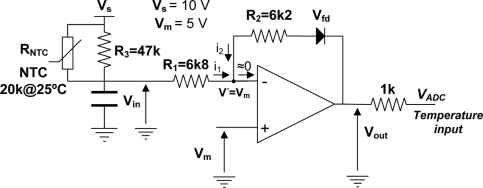
Signal conditioning circuit for the embedded temperature sensor (NTC).

**Figure 9.**
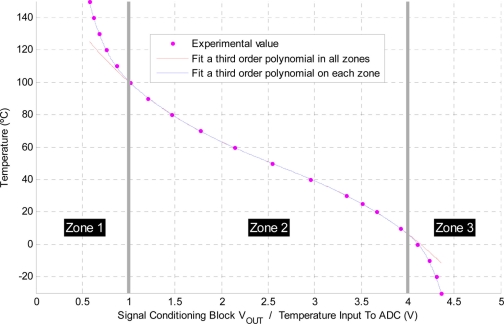
Temperature *vs.* Output voltage after signal conditioning.

The temperature signal conditioning circuit is shown Figure 8. The input voltage is determined using Equation (3) whilst Equation (4) corresponds to the output voltage. Considering the circuit shown in Figure 8, without the diode in the feedback loop (V_fd_ = 0) and using the following resistor values R1 = 6k8, R2 = 6k2, R3 = 47k the characteristic curve that relates temperature and output voltage is shown in Figure 9. Experimental data is curve fitted using the third order polynomial as shown in Table 4.

(3) $$V_{\mathrm{in}}=i_{1}\cdot R_{1}=\frac{V_{S}-V_{m}}{(R_{\mathrm{NTC}}∥R_{3})+R_{1}}R_{1}$$

(4) $$V_{\mathrm{out}}=\frac{R_{2}V_{\mathrm{in}}+R_{1}V_{\mathrm{fd}}-(R_{2}+R_{1})V_{m}}{-R_{1}}$$

Three different zones are identified depending on the input voltage. The selection of a third order polynomial is chosen as it can be implemented in a low cost MCU providing good temperature accuracy. The final temperature accuracy is within ±0.5 °C within a working temperature range of −20 to 150 °C.

#### 2.2.2. Marginal Oscillator Circuit

A marginal oscillator is a resonant oscillator whose operating level is controlled by the non-linearity of the transfer characteristic of the oscillator gain element. Marginal oscillators respond to small changes in tuned circuit resistance with a change in oscillation amplitude whilst changes in tuned circuit reactance cause a shift in the frequency of oscillation [35].

#### 2.2.2.1. Frequency of Oscillation

**Figure 10.**
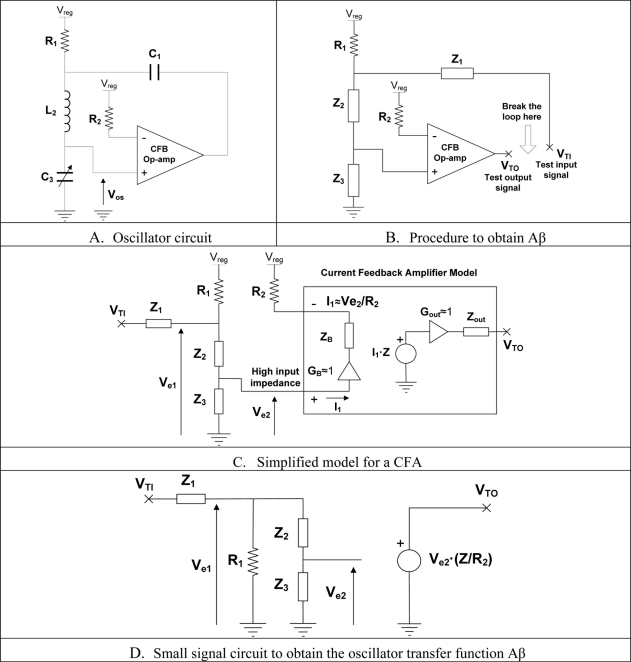
Simplified CFA model to obtain the oscillation condition.

The oscillator circuit is shown in Figure 10. The transfer function of the product of amplifier and feedback circuit is obtained from Figure 10(D). The transfer function of the circuit (Aβ) is shown in Equation (5). The oscillation condition is obtained using the simplified small signal model, Figure 10(D). According to Nyquist criterion, the oscillation condition is achieved when the phase of Aβ is 0 and the gain is greater than 1 at the oscillation frequency ω_osc_ Using the simplified CFA model [36], the oscillation condition is determined for pure reactive elements, Equation (6). As it can be seen the oscillation condition is imposed by the inductor and sensing capacitor. Therefore to satisfy this condition, the term X_2_ + X_3_ should be 0 in equation to make Aβ real.

(5) $$\frac{V_{\mathrm{TO}}}{V_{\mathrm{TI}}}=\frac{Z_{3}[\frac{R_{1}\cdot Z}{R_{2}}]}{(R_{1}+Z_{2}+Z_{3})Z_{1}+(Z_{2}+Z_{3})R_{1}}G=\frac{R_{1}\cdot Z}{R_{2}}$$

Considering pure reactive elements, the transfer function is reduced to Equation (6):

(6) $$\begin{array}{c} Z_{1}={\mathrm{jX}}_{1},Z_{2}={\mathrm{jX}}_{2},Z_{3}={\mathrm{jX}}_{3}\\ A\beta =\frac{V_{\mathrm{TO}}}{V_{\mathrm{TI}}}=\frac{{\mathrm{jX}}_{3}[\frac{R_{1}\cdot Z}{R_{2}}]}{(R_{1}+{\mathrm{jX}}_{2}+{\mathrm{jX}}_{3}){\mathrm{jX}}_{1}+({\mathrm{jX}}_{2}+{\mathrm{jX}}_{3})R_{1}} \end{array}$$

Imposing the oscillation condition in Equation (6), the following relationship is obtained, Equation (7):

(7) $$\begin{array}{c} A\beta =0{}^{\circ}\to X_{2}(\omega_{\mathrm{OSC}})+X_{3}(\omega_{\mathrm{OSC}})=0\\ A\beta =\frac{V_{\mathrm{TO}}}{V_{\mathrm{TI}}}=\frac{{\mathrm{jX}}_{3}(\omega_{\mathrm{OSC}})[\frac{R_{1}\cdot Z}{R_{2}}]}{{\mathrm{jX}}_{1}(\omega_{\mathrm{OSC}})R_{1}}=\frac{X_{3}(\omega_{\mathrm{OSC}})[\frac{Z}{R_{2}}]}{X_{1}(\omega_{\mathrm{OSC}})}>1 \end{array}$$

The frequency of oscillation when X_2_ is an inductor and X_3_ a capacitor is fixed by Equation (8):

(8) $$\omega_{\mathrm{OSC}}=\frac{1}{2\pi \sqrt{L_{2}C_{3}}}$$

##### 2.2.2.2. Criteria for Selecting the Optimum Oscillation Frequency

The sensor can be tuned at any frequency within the 1 to 100 MHz range. However, the optimum design should be chosen considering the lowest measurement frequency (to gain more sensitivity) that fulfills the requirement of impedance reading variations due to oscillator frequency drifts (e.g., ΔZ < 1%). Frequency drifts are due to changes in the reactive elements as shown in Equation (8). Therefore, the inductor and the capacitors for the oscillator circuit should have a very low temperature coefficient. Ceramic chip inductors are suitable for this application as they show very low core losses, high Q values and relatively high inductance stability over the operating temperature ranges.

The optimum frequency selection can be graphically chosen from the frequency sweeps of the dissipation factor bearing in mind that tan δ is directly related to the complex impedance, Figure 2. For example, Figure 4 shows the frequency sweeps of the dissipation factor for hydraulic oil without and with water contamination respectively. Possible candidates for the best operational frequencies seem to be around 1 MHz where tan δ has almost the same value within a 1 MHz span and 13 MHz where tan δ is almost constant for oscillator frequency drifts. The lower frequency is optimum because the tan δ sensitivity of oil degradation is better than the 13 MHz region. As a result, considering these two observations the best frequency to perform measurements is around the MHz region.

##### 2.2.2.3. Signal Condition of the Oscillation Peak Amplitude

**Figure 11.**
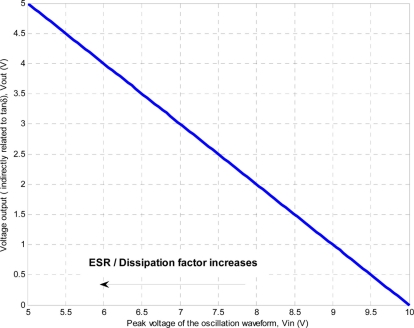
Output voltage proportional to tan δ depending on the peak voltage of the oscillation waveform.

The amplitude of oscillation is buffered using a unity gain non-inverter CFA. The buffered signal passes through a peak detection circuit which consists in a diode and one capacitor. This circuit stores a voltage proportional to the peak of oscillation. A further stage is required to translate the peak amplitude to MCU voltage levels (0 to 5 V). The amplifier inverts the input signal and performs voltage translation in such way that an increase of output signal corresponds to an increase of ESR or dissipation factor. The selected stage also compensates the peak detector circuit voltage drop due to the forward voltage of the diode which is temperature dependant. The governing equation of the whole peak amplitude detection circuit is plot in Figure 11.

## 3. Results and Discussion

The oscillator circuit of Figure 10(A) was simulated using ISIS v7.6 distributed by Labcenter Electronics. The PSPICE amplifier model used by the simulator is more realistic than the simplified one used to obtain the frequency of oscillation, therefore obtained results are closer to real circuit operation. Simulation helps to understand the effect of R_1_, L_2_, C_1_, C_3_ in the voltage peak of oscillation and oscillation frequency. The following list of components were used: the CFA is AD8001, R1 is 2 Ω, R2 is 300 Ω, C1 is 820 pF and C3 is the equivalent load. The following sine wave parameters were obtained for different equivalent sensor loads, Table 5. The sensitivity of the circuit is not linearly dependant with the range of ESR. The circuit can detect small changes of ESR with excellent resolution between 1 to 10 Ω which corresponds to the sensor output voltage within the range 0 to 3 V. From 10 to 100 Ω the sensitivity is much lower which corresponds to the sensor output voltage within the range 4 to 4.5 V and finally from 100 Ω onwards the sensors sensitivity is very coarse.

**Figure 12.**
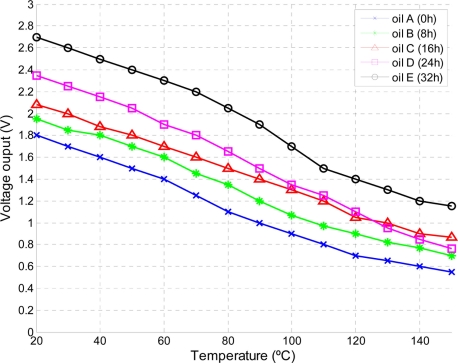
Sensor’s output voltage *vs.* temperature for different oil oxidation stages.

Lubricant samples were prepared under specific oxidation conditions. Air was pump into a vessel containing lubricant. The temperature of the vessel is controlled using a heating bath with PID temperature control. Copper wire catalyst is used to accelerate the oxidation process. Five samples at different oxidation stages were obtained exposing an oil of grade 15W/40 under 150 °C for 0, 8, 16, 24, 32 h. After sample preparation the output of the sensor was recorded for a range of temperatures within 20 °C to 150 °C. Results are shown in Figure 12.

**Figure 13.**
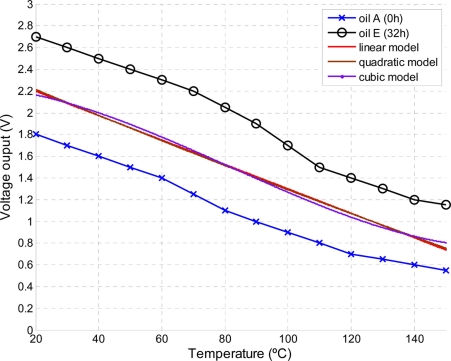
Regression models to extract the temperature dependency. (All samples were considered, Oil A,B,C,D,E)

At a given temperature the output voltage is proportional to the oxidation stage. The experimental data in Figure 12 is curve fitted to different regression models in order to obtain the temperature dependency of the sensor’s output. As shown in Figure 13, a linear model fits the data (5 datasets) with a good degree of accuracy (m = −0.01123, b = 2.422). The selected model can be implemented in a MCU to compensate temperature drifts due to temperature variations.

The same procedure for temperature compensation can be applied to a specific machine lubricant. The experimental dataset consists on lubricant samples at different degradation stages and sampling time intervals. As a result, a custom based calibration approach could be performed providing a more meaningful indication of the oil condition for a specific industry asset.

## 4. Conclusions

Impedance monitoring of lubricants is an important tool for detecting the condition of oils. A low cost oil quality sensor based on a marginal oscillator is presented which monitors changes on the impedance of the sensing electrodes at high frequencies. The circuit provides a voltage output related to the dissipation factor and therefore, it provides an indication of the oil condition as the dissipation factor tends to increase with the increasing presence of contaminants in lubrication oil. The proposed sensor shows three important features: it is a very low cost design, it can be custom calibrated for a specific lubricant and it provides effective oil quality detection. This conclusion means that this sensor is an attractive alternative compared with other types of oil condition sensors.

## Figures and Tables

**Table 1.** Capacitance for different electrode geometries.

$C=\frac{Q}{V}=\frac{{Aɛ}_{0}{{ɛ}^{\prime }}_{r}}{D}$	**Capacitance for parallel plates (F)**
	A→Electrode’s area (m^2^)
	D→Distance between electrodes (m)
$C=\frac{Q}{V}=\frac{{2\pi ɛ}_{0}{{ɛ}^{\prime }}_{r}L}{\text{ln}(\frac{b}{a})}$	**Capacitance for a cylindrical capacitor (F)**
	L→length of the cylinder (m)
	a→radius of the inner cylinder (m)
	b→radius of the outer cylinder (m)

**Table 2.** Geometrical dimensions of sensor electrodes and expected capacitance.

**Cylindrical head**	**L (mm)**	**b (mm)**	**a (mm)**	**C_0_ (pF), ɛ_0_**	**C (pF), (ɛ_r_ = 2.25)**	**C (pF), (ɛ_r_ = 2.45)**
	12	8	7	50	112	122

**Table 3.** Steinhart coefficients for the NTC.

**b_0_**	**b_1_**	**b_3_**
1.379 × 10^−3^	1.780 × 10^−4^	2.206 × 10^−7^

**Table 4.** Curve fitting coefficients for the temperature signal conditioning circuit.

**Zone**	**Third order polynomial (p_1_·x^3^ + p_2_·x^2^ + p_3_·x + p_4_)**			
	**p_1_**	**p_2_**	**p_3_**	**p_4_**

**1^st^** → (0–1)V	−241	765.9	−861.5	437.9
**2^nd^** → (1–4)V	−3.6	28.1	−96.1	172
**3^rd^** → (4–5)V	−212	2464	−9580	12,469
**All zones**	−3.6	28.1	−96.1	172

**Table 5.** Simulation results for the equivalent sensing circuit.

**Equivalent load, sensing head**		**Peak of voltage oscillation (V)**
**ESR (Ω)**	**C (pF)**	

1	40 (air ɛ_0_)	6.1
1	112	>10 (CFA is saturated)
1	116	>10 (CFA is saturated)
1	120	>10 (CFA is saturated)
2.5	120	9.0
5	120	8.1
7.5	120	7.4
10	112	7.1
10	116	7.1
10	120	7.1
25	120	6.1
50	120	5.7
75	120	5.5
100	112	5.4
100	116	5.4
100	120	5.4
